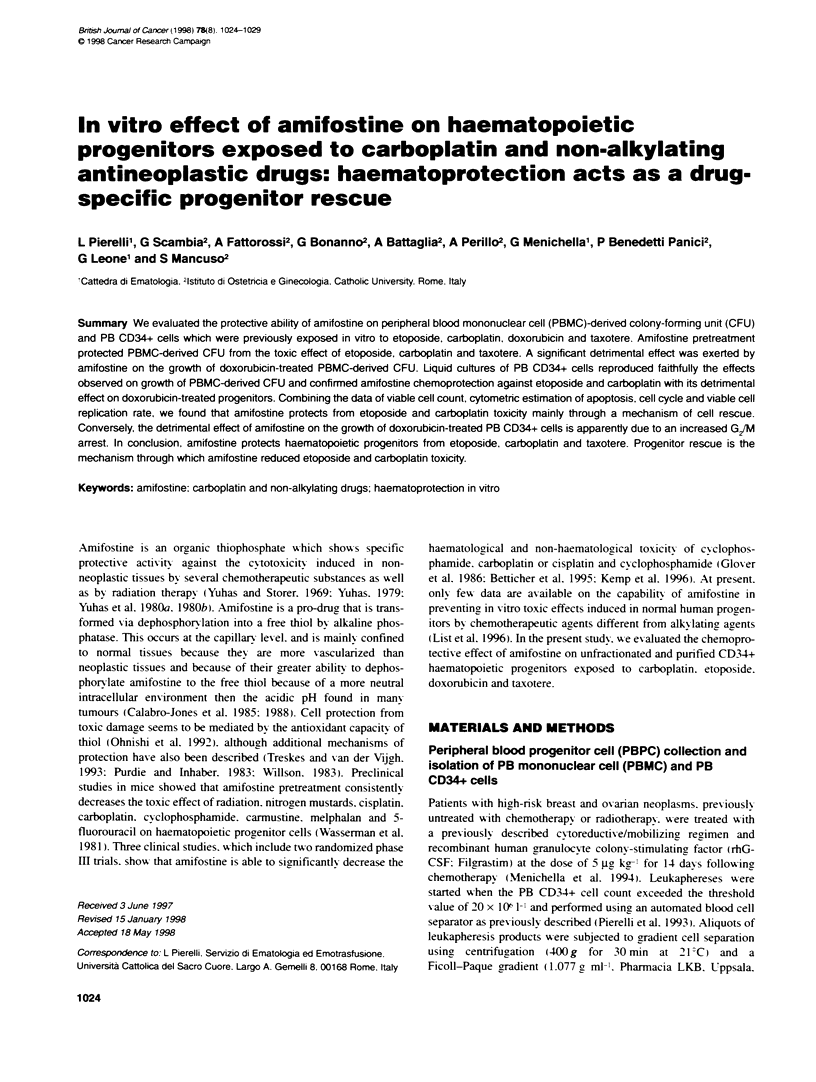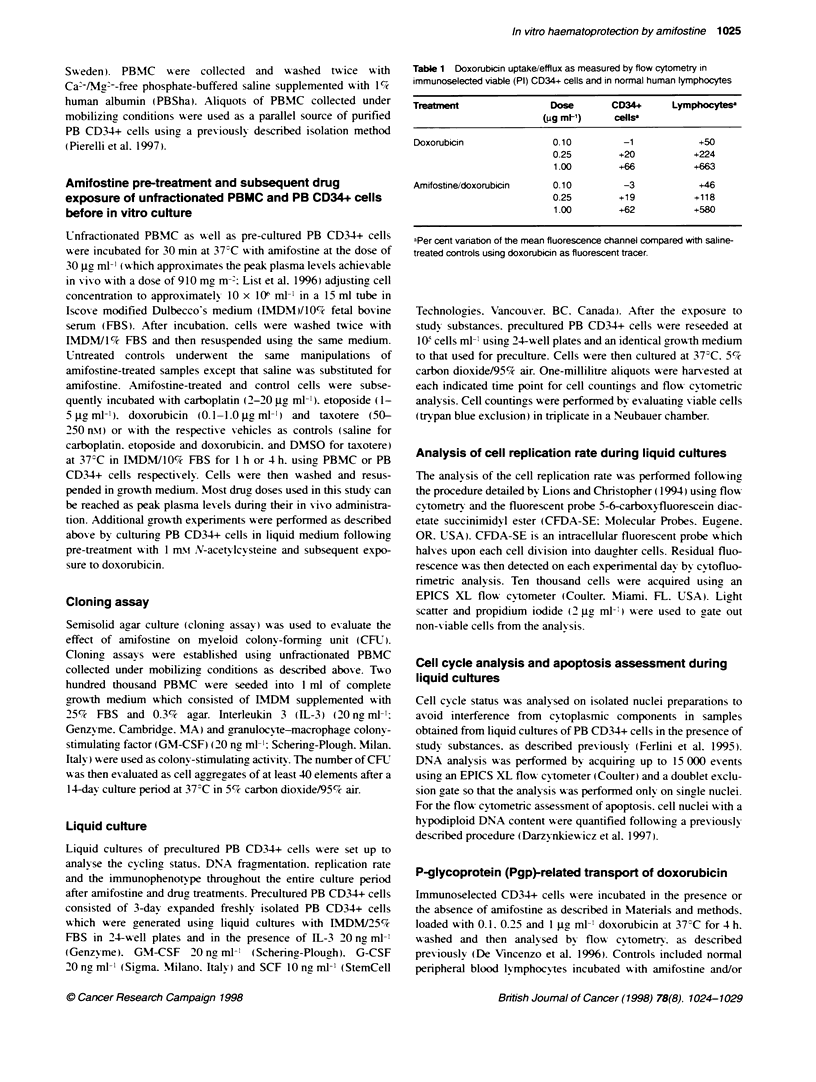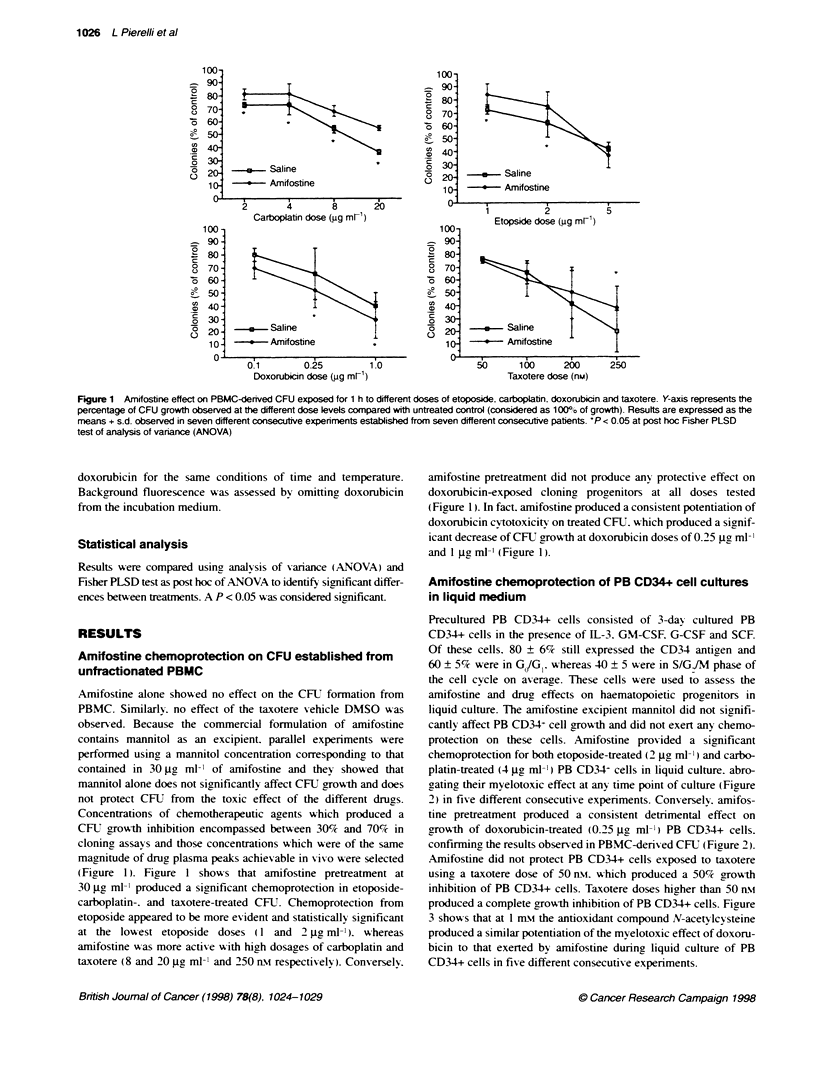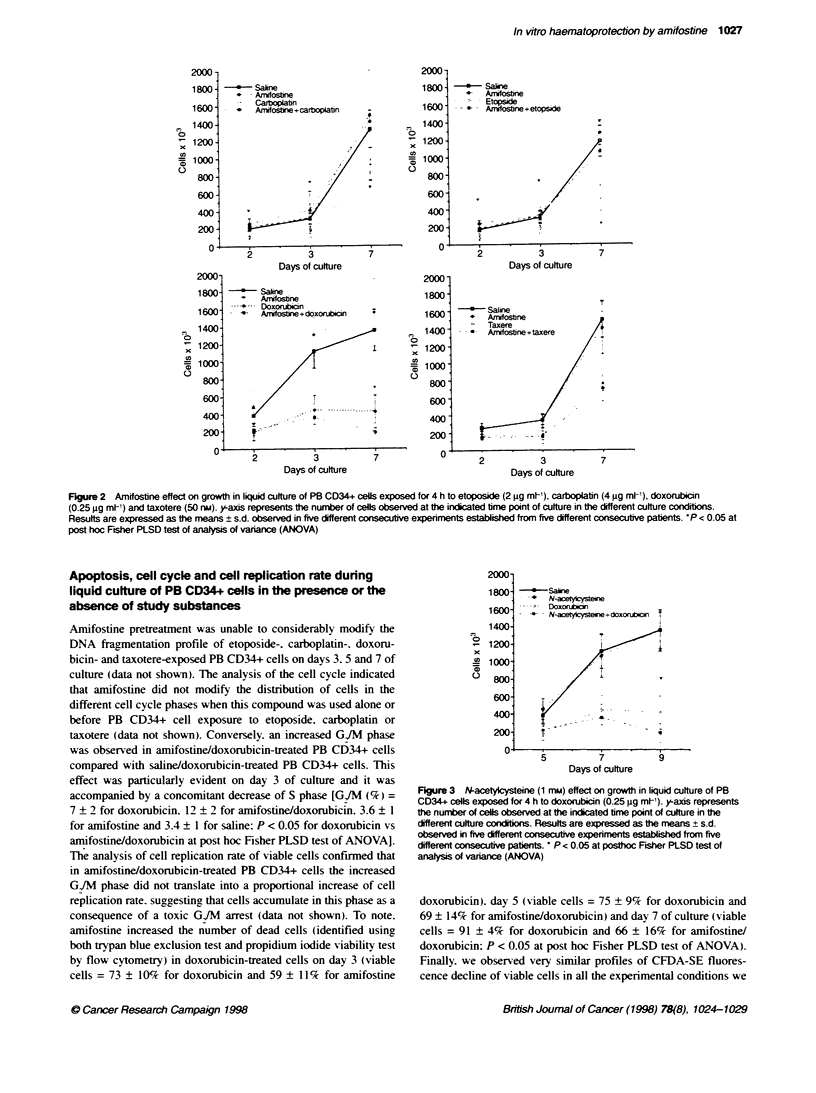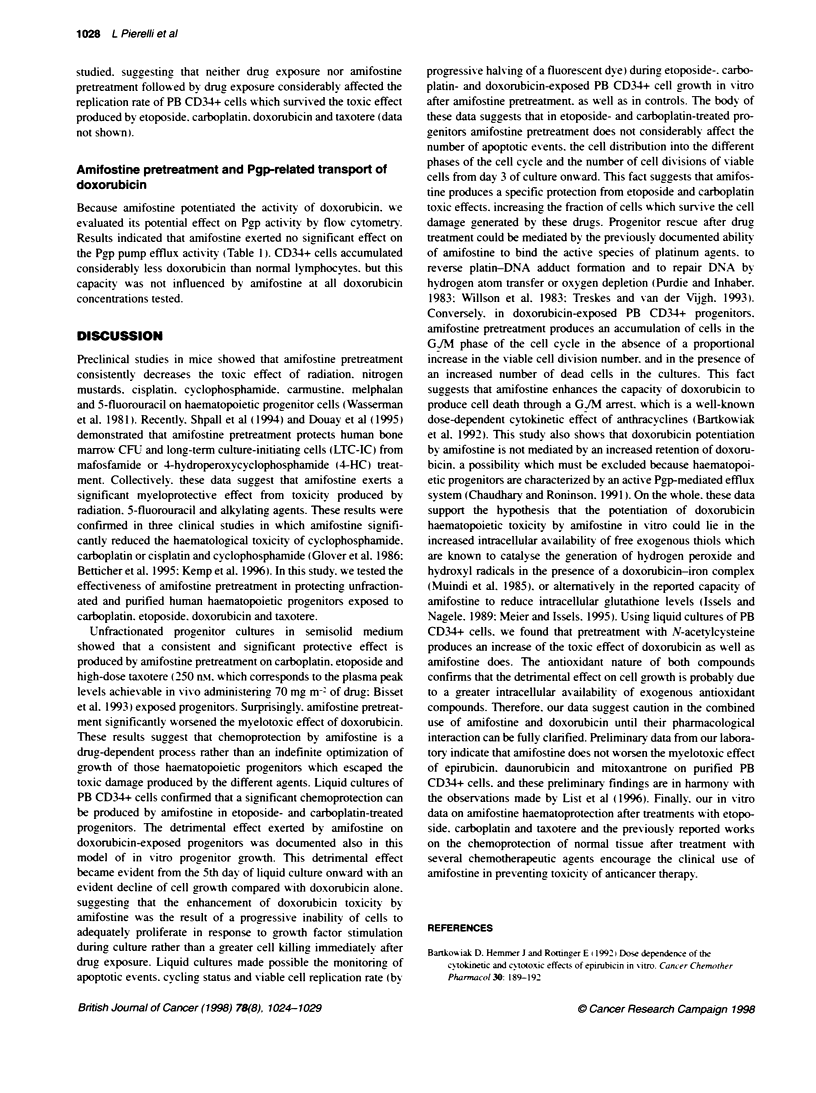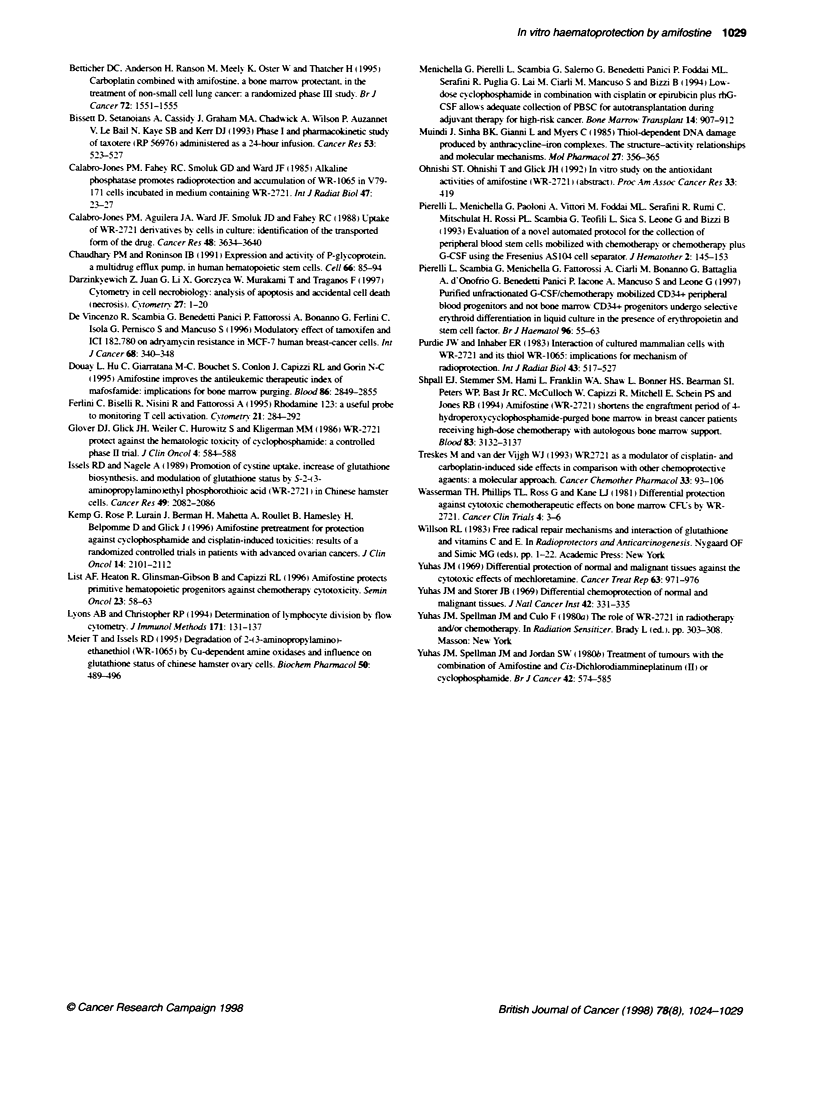# In vitro effect of amifostine on haematopoietic progenitors exposed to carboplatin and non-alkylating antineoplastic drugs: haematoprotection acts as a drug-specific progenitor rescue.

**DOI:** 10.1038/bjc.1998.622

**Published:** 1998-10

**Authors:** L. Pierelli, G. Scambia, A. Fattorossi, G. Bonanno, A. Battaglia, A. Perillo, G. Menichella, P. B. Panici, G. Leone, S. Mancuso

**Affiliations:** Cattedra di Ematologia, Catholic University, Rome, Italy.

## Abstract

We evaluated the protective ability of amifostine on peripheral blood mononuclear cell (PBMC)-derived colony-forming unit (CFU) and PB CD34+ cells which were previously exposed in vitro to etoposide, carboplatin, doxorubicin and taxotere. Amifostine pretreatment protected PBMC-derived CFU from the toxic effect of etoposide, carboplatin and taxotere. A significant detrimental effect was exerted by amifostine on the growth of doxorubicin-treated PBMC-derived CFU. Liquid cultures of PB CD34+ cells reproduced faithfully the effects observed on growth of PBMC-derived CFU and confirmed amifostine chemoprotection against etoposide and carboplatin with its detrimental effect on doxorubicin-treated progenitors. Combining the data of viable cell count, cytometric estimation of apoptosis, cell cycle and viable cell replication rate, we found that amifostine protects from etoposide and carboplatin toxicity mainly through a mechanism of cell rescue. Conversely, the detrimental effect of amifostine on the growth of doxorubicin-treated PB CD34+ cells is apparently due to an increased G2/M arrest. In conclusion, amifostine protects haematopoietic progenitors from etoposide, carboplatin and taxotere. Progenitor rescue is the mechanism through which amifostine reduced etoposide and carboplatin toxicity.


British Journal of Cancer (1998) 78(8), 1024–1029
© 1998 Cancer Research Campaign

### In vitro effect of amifostine on haematopoietic progenitors exposed to carboplatin and non-alkylating antineoplastic drugs: haematoprotection acts as a drug-specific progenitor rescue

L Pierelli[1], G Scambia[2], A Fattorossi[2], G Bonanno[2], A Battaglia[2], A Perillo[2], G Menichella[1], P Benedetti Panici[2], G Leone[1] and S Mancuso[2]

[1]Cattedra di Ematologia. [2]Istituto di Ostetricia e Ginecologia. Catholic University. Rome. Italy

**Summary** We evaluated the protective ability of amifostine on peripheral blood mononuclear cell (PBMC)-derived colony-forming unit (CFU) and PB CD34+ cells which were previously exposed in vitro to etoposide, carboplatin, doxorubicin and taxotere. Amifostine pretreatment protected PBMC-derived CFU from the toxic effect of etoposide, carboplatin and taxotere. A significant detrimental effect was exerted by amifostine on the growth of doxorubicin-treated PBMC-derived CFU. Liquid cultures of PB CD34+ cells reproduced faithfully the effects observed on growth of PBMC-derived CFU and confirmed amifostine chemoprotection against etoposide and carboplatin with its detrimental effect on doxorubicin-treated progenitors. Combining the data of viable cell count, cytometric estimation of apoptosis, cell cycle and viable cell replication rate, we found that amifostine protects from etoposide and carboplatin toxicity mainly through a mechanism of cell rescue. Conversely, the detrimental effect of amifostine on the growth of doxorubicin-treated PB CD34+ cells is apparently due to an increased $G_2/M$ arrest. In conclusion, amifostine protects haematopoietic progenitors from etoposide, carboplatin and taxotere. Progenitor rescue is the mechanism through which amifostine reduced etoposide and carboplatin toxicity.

**Keywords:** amifostine; carboplatin and non-alkylating drugs; haematoprotection in vitro

Amifostine is an organic thiophosphate which shows specific protective activity against the cytotoxicity induced in non-neoplastic tissues by several chemotherapeutic substances as well as by radiation therapy (Yuhas and Storer, 1969; Yuhas, 1979; Yuhas et al, 1980*a*, 1980*b*). Amifostine is a pro-drug that is transformed via dephosphorylation into a free thiol by alkaline phosphatase. This occurs at the capillary level, and is mainly confined to normal tissues because they are more vascularized than neoplastic tissues and because of their greater ability to dephosphorylate amifostine to the free thiol because of a more neutral intracellular environment then the acidic pH found in many tumours (Calabro-Jones et al, 1985; 1988). Cell protection from toxic damage seems to be mediated by the antioxidant capacity of thiol (Ohnishi et al, 1992), although additional mechanisms of protection have also been described (Treskes and van der Vijgh, 1993; Purdie and Inhaber, 1983; Willson, 1983). Preclinical studies in mice showed that amifostine pretreatment consistently decreases the toxic effect of radiation, nitrogen mustards, cisplatin, carboplatin, cyclophosphamide, carmustine, melphalan and 5-fluorouracil on haematopoietic progenitor cells (Wasserman et al, 1981). Three clinical studies, which include two randomized phase III trials, show that amifostine is able to significantly decrease the haematological and non-haematological toxicity of cyclophosphamide, carboplatin or cisplatin and cyclophosphamide (Glover et al, 1986; Betticher et al, 1995; Kemp et al, 1996). At present, only few data are available on the capability of amifostine in preventing in vitro toxic effects induced in normal human progenitors by chemotherapeutic agents different from alkylating agents (List et al, 1996). In the present study, we evaluated the chemoprotective effect of amifostine on unfractionated and purified CD34+ haematopoietic progenitors exposed to carboplatin, etoposide, doxorubicin and taxotere.

*Received 3 June 1997*
*Revised 15 January 1998*
*Accepted 18 May 1998*

*Correspondence to:* L Pierelli, Servizio di Ematologia ed Emotrasfusione, Università Cattolica del Sacro Cuore, Largo A. Gemelli 8, 00168 Rome, Italy

### MATERIALS AND METHODS

### Peripheral blood progenitor cell (PBPC) collection and isolation of PB mononuclear cell (PBMC) and PB CD34+ cells

Patients with high-risk breast and ovarian neoplasms, previously untreated with chemotherapy or radiotherapy, were treated with a previously described cytoreductive/mobilizing regimen and recombinant human granulocyte colony-stimulating factor (rhG-CSF; Filgrastim) at the dose of 5 μg $kg^{-1}$ for 14 days following chemotherapy (Menichella et al, 1994). Leukaphereses were started when the PB CD34+ cell count exceeded the threshold value of $20 \times 10^6\ l^{-1}$ and performed using an automated blood cell separator as previously described (Pierelli et al, 1993). Aliquots of leukapheresis products were subjected to gradient cell separation using centrifugation (400 *g* for 30 min at 21°C) and a Ficoll–Paque gradient (1.077 g $ml^{-1}$, Pharmacia LKB, Uppsala,

Sweden). PBMC were collected and washed twice with $Ca^{2+}/Mg^{2+}$-free phosphate-buffered saline supplemented with 1% human albumin (PBSha). Aliquots of PBMC collected under mobilizing conditions were used as a parallel source of purified PB CD34+ cells using a previously described isolation method (Pierelli et al. 1997).

### Amifostine pre-treatment and subsequent drug exposure of unfractionated PBMC and PB CD34+ cells before in vitro culture

Unfractionated PBMC as well as pre-cultured PB CD34+ cells were incubated for 30 min at 37°C with amifostine at the dose of 30 μg $ml^{-1}$ (which approximates the peak plasma levels achievable in vivo with a dose of 910 mg $m^{-2}$: List et al. 1996) adjusting cell concentration to approximately $10 \times 10^6$ $ml^{-1}$ in a 15 ml tube in Iscove modified Dulbecco's medium (IMDM)/10% fetal bovine serum (FBS). After incubation, cells were washed twice with IMDM/1% FBS and then resuspended using the same medium. Untreated controls underwent the same manipulations of amifostine-treated samples except that saline was substituted for amifostine. Amifostine-treated and control cells were subsequently incubated with carboplatin (2–20 μg $ml^{-1}$), etoposide (1–5 μg $ml^{-1}$), doxorubicin (0.1–1.0 μg $ml^{-1}$) and taxotere (50–250 nM) or with the respective vehicles as controls (saline for carboplatin, etoposide and doxorubicin, and DMSO for taxotere) at 37°C in IMDM/10% FBS for 1 h or 4 h, using PBMC or PB CD34+ cells respectively. Cells were then washed and resuspended in growth medium. Most drug doses used in this study can be reached as peak plasma levels during their in vivo administration. Additional growth experiments were performed as described above by culturing PB CD34+ cells in liquid medium following pre-treatment with 1 mM *N*-acetylcysteine and subsequent exposure to doxorubicin.

### Cloning assay

Semisolid agar culture (cloning assay) was used to evaluate the effect of amifostine on myeloid colony-forming unit (CFU). Cloning assays were established using unfractionated PBMC collected under mobilizing conditions as described above. Two hundred thousand PBMC were seeded into 1 ml of complete growth medium which consisted of IMDM supplemented with 25% FBS and 0.3% agar. Interleukin 3 (IL-3) (20 ng $ml^{-1}$: Genzyme, Cambridge, MA) and granulocyte–macrophage colony-stimulating factor (GM-CSF) (20 ng $ml^{-1}$: Schering-Plough, Milan, Italy) were used as colony-stimulating activity. The number of CFU was then evaluated as cell aggregates of at least 40 elements after a 14-day culture period at 37°C in 5% carbon dioxide/95% air.

### Liquid culture

Liquid cultures of precultured PB CD34+ cells were set up to analyse the cycling status, DNA fragmentation, replication rate and the immunophenotype throughout the entire culture period after amifostine and drug treatments. Precultured PB CD34+ cells consisted of 3-day expanded freshly isolated PB CD34+ cells which were generated using liquid cultures with IMDM/25% FBS in 24-well plates and in the presence of IL-3 20 ng $ml^{-1}$ (Genzyme), GM-CSF 20 ng $ml^{-1}$ (Schering-Plough), G-CSF 20 ng $ml^{-1}$ (Sigma, Milano, Italy) and SCF 10 ng $ml^{-1}$ (StemCell Technologies, Vancouver, BC, Canada). After the exposure to study substances, precultured PB CD34+ cells were reseeded at $10^5$ cells $ml^{-1}$ using 24-well plates and an identical growth medium to that used for preculture. Cells were then cultured at 37°C, 5% carbon dioxide/95% air. One-millilitre aliquots were harvested at each indicated time point for cell countings and flow cytometric analysis. Cell countings were performed by evaluating viable cells (trypan blue exclusion) in triplicate in a Neubauer chamber.

**Table 1** Doxorubicin uptake/efflux as measured by flow cytometry in immunoselected viable (PI) CD34+ cells and in normal human lymphocytes

| Treatment | Dose (μg $ml^{-1}$) | CD34+ cells[a] | Lymphocytes[a] |
|---|---|---|---|
| Doxorubicin | 0.10 | −1 | +50 |
| | 0.25 | +20 | +224 |
| | 1.00 | +66 | +663 |
| Amifostine/doxorubicin | 0.10 | −3 | +46 |
| | 0.25 | +19 | +118 |
| | 1.00 | +62 | +580 |

[a]Per cent variation of the mean fluorescence channel compared with saline-treated controls using doxorubicin as fluorescent tracer.

### Analysis of cell replication rate during liquid cultures

The analysis of the cell replication rate was performed following the procedure detailed by Lions and Christopher (1994) using flow cytometry and the fluorescent probe 5-6-carboxyfluorescein diacetate succinimidyl ester (CFDA-SE: Molecular Probes, Eugene, OR, USA). CFDA-SE is an intracellular fluorescent probe which halves upon each cell division into daughter cells. Residual fluorescence was then detected on each experimental day by cytofluorimetric analysis. Ten thousand cells were acquired using an EPICS XL flow cytometer (Coulter, Miami, FL, USA). Light scatter and propidium iodide (2 μg $ml^{-1}$) were used to gate out non-viable cells from the analysis.

### Cell cycle analysis and apoptosis assessment during liquid cultures

Cell cycle status was analysed on isolated nuclei preparations to avoid interference from cytoplasmic components in samples obtained from liquid cultures of PB CD34+ cells in the presence of study substances, as described previously (Ferlini et al. 1995). DNA analysis was performed by acquiring up to 15 000 events using an EPICS XL flow cytometer (Coulter) and a doublet exclusion gate so that the analysis was performed only on single nuclei. For the flow cytometric assessment of apoptosis, cell nuclei with a hypodiploid DNA content were quantified following a previously described procedure (Darzynkiewicz et al. 1997).

### P-glycoprotein (Pgp)-related transport of doxorubicin

Immunoselected CD34+ cells were incubated in the presence or the absence of amifostine as described in Materials and methods, loaded with 0.1, 0.25 and 1 μg $ml^{-1}$ doxorubicin at 37°C for 4 h, washed and then analysed by flow cytometry, as described previously (De Vincenzo et al. 1996). Controls included normal peripheral blood lymphocytes incubated with amifostine and/or

© Cancer Research Campaign 1998

**Figure 1** Amifostine effect on PBMC-derived CFU exposed for 1 h to different doses of etoposide, carboplatin, doxorubicin and taxotere. *Y*-axis represents the percentage of CFU growth observed at the different dose levels compared with untreated control (considered as 100% of growth). Results are expressed as the means + s.d. observed in seven different consecutive experiments established from seven different consecutive patients. *$P < 0.05$ at post hoc Fisher PLSD test of analysis of variance (ANOVA)

doxorubicin for the same conditions of time and temperature. Background fluorescence was assessed by omitting doxorubicin from the incubation medium.

### Statistical analysis

Results were compared using analysis of variance (ANOVA) and Fisher PLSD test as post hoc of ANOVA to identify significant differences between treatments. A $P < 0.05$ was considered significant.

### RESULTS

### Amifostine chemoprotection on CFU established from unfractionated PBMC

Amifostine alone showed no effect on the CFU formation from PBMC. Similarly, no effect of the taxotere vehicle DMSO was observed. Because the commercial formulation of amifostine contains mannitol as an excipient, parallel experiments were performed using a mannitol concentration corresponding to that contained in 30 μg ml$^{-1}$ of amifostine and they showed that mannitol alone does not significantly affect CFU growth and does not protect CFU from the toxic effect of the different drugs. Concentrations of chemotherapeutic agents which produced a CFU growth inhibition encompassed between 30% and 70% in cloning assays and those concentrations which were of the same magnitude of drug plasma peaks achievable in vivo were selected (Figure 1). Figure 1 shows that amifostine pretreatment at 30 μg ml$^{-1}$ produced a significant chemoprotection in etoposide-carboplatin-, and taxotere-treated CFU. Chemoprotection from etoposide appeared to be more evident and statistically significant at the lowest etoposide doses (1 and 2 μg ml$^{-1}$), whereas amifostine was more active with high dosages of carboplatin and taxotere (8 and 20 μg ml$^{-1}$ and 250 nM respectively). Conversely, amifostine pretreatment did not produce any protective effect on doxorubicin-exposed cloning progenitors at all doses tested (Figure 1). In fact, amifostine produced a consistent potentiation of doxorubicin cytotoxicity on treated CFU, which produced a significant decrease of CFU growth at doxorubicin doses of 0.25 μg ml$^{-1}$ and 1 μg ml$^{-1}$ (Figure 1).

### Amifostine chemoprotection of PB CD34+ cell cultures in liquid medium

Precultured PB CD34+ cells consisted of 3-day cultured PB CD34+ cells in the presence of IL-3, GM-CSF, G-CSF and SCF. Of these cells, 80 ± 6% still expressed the CD34 antigen and 60 ± 5% were in $G_0/G_1$, whereas 40 ± 5 were in $S/G_2/M$ phase of the cell cycle on average. These cells were used to assess the amifostine and drug effects on haematopoietic progenitors in liquid culture. The amifostine excipient mannitol did not significantly affect PB $CD34^+$ cell growth and did not exert any chemoprotection on these cells. Amifostine provided a significant chemoprotection for both etoposide-treated (2 μg ml$^{-1}$) and carboplatin-treated (4 μg ml$^{-1}$) PB $CD34^+$ cells in liquid culture, abrogating their myelotoxic effect at any time point of culture (Figure 2) in five different consecutive experiments. Conversely, amifostine pretreatment produced a consistent detrimental effect on growth of doxorubicin-treated (0.25 μg ml$^{-1}$) PB CD34+ cells, confirming the results observed in PBMC-derived CFU (Figure 2). Amifostine did not protect PB CD34+ cells exposed to taxotere using a taxotere dose of 50 nM, which produced a 50% growth inhibition of PB CD34+ cells. Taxotere doses higher than 50 nM produced a complete growth inhibition of PB CD34+ cells. Figure 3 shows that at 1 mM the antioxidant compound *N*-acetylcysteine produced a similar potentiation of the myelotoxic effect of doxorubicin to that exerted by amifostine during liquid culture of PB CD34+ cells in five different consecutive experiments.

 © Cancer Research Campaign 1998

**Figure 2** Amifostine effect on growth in liquid culture of PB CD34+ cells exposed for 4 h to etoposide (2 μg ml⁻¹), carboplatin (4 μg ml⁻¹), doxorubicin (0.25 μg ml⁻¹) and taxotere (50 nM). *y*-axis represents the number of cells observed at the indicated time point of culture in the different culture conditions. Results are expressed as the means ± s.d. observed in five different consecutive experiments established from five different consecutive patients. *$P < 0.05$ at post hoc Fisher PLSD test of analysis of variance (ANOVA)

### Apoptosis, cell cycle and cell replication rate during liquid culture of PB CD34+ cells in the presence or the absence of study substances

Amifostine pretreatment was unable to considerably modify the DNA fragmentation profile of etoposide-, carboplatin-, doxorubicin- and taxotere-exposed PB CD34+ cells on days 3, 5 and 7 of culture (data not shown). The analysis of the cell cycle indicated that amifostine did not modify the distribution of cells in the different cell cycle phases when this compound was used alone or before PB CD34+ cell exposure to etoposide, carboplatin or taxotere (data not shown). Conversely, an increased $G_2/M$ phase was observed in amifostine/doxorubicin-treated PB CD34+ cells compared with saline/doxorubicin-treated PB CD34+ cells. This effect was particularly evident on day 3 of culture and it was accompanied by a concomitant decrease of S phase [$G_2/M$ (%) = 7 ± 2 for doxorubicin, 12 ± 2 for amifostine/doxorubicin, 3.6 ± 1 for amifostine and 3.4 ± 1 for saline: $P < 0.05$ for doxorubicin vs amifostine/doxorubicin at post hoc Fisher PLSD test of ANOVA]. The analysis of cell replication rate of viable cells confirmed that in amifostine/doxorubicin-treated PB CD34+ cells the increased $G_2/M$ phase did not translate into a proportional increase of cell replication rate, suggesting that cells accumulate in this phase as a consequence of a toxic $G_2/M$ arrest (data not shown). To note, amifostine increased the number of dead cells (identified using both trypan blue exclusion test and propidium iodide viability test by flow cytometry) in doxorubicin-treated cells on day 3 (viable cells = 73 ± 10% for doxorubicin and 59 ± 11% for amifostine

**Figure 3** *N*-acetylcysteine (1 mM) effect on growth in liquid culture of PB CD34+ cells exposed for 4 h to doxorubicin (0.25 μg ml⁻¹). *y*-axis represents the number of cells observed at the indicated time point of culture in the different culture conditions. Results are expressed as the means ± s.d. observed in five different consecutive experiments established from five different consecutive patients. * $P < 0.05$ at posthoc Fisher PLSD test of analysis of variance (ANOVA)

doxorubicin), day 5 (viable cells = 75 ± 9% for doxorubicin and 69 ± 14% for amifostine/doxorubicin) and day 7 of culture (viable cells = 91 ± 4% for doxorubicin and 66 ± 16% for amifostine/doxorubicin: $P < 0.05$ at post hoc Fisher PLSD test of ANOVA). Finally, we observed very similar profiles of CFDA-SE fluorescence decline of viable cells in all the experimental conditions we

© Cancer Research Campaign 1998

studied, suggesting that neither drug exposure nor amifostine pretreatment followed by drug exposure considerably affected the replication rate of PB CD34+ cells which survived the toxic effect produced by etoposide, carboplatin, doxorubicin and taxotere (data not shown).

### Amifostine pretreatment and Pgp-related transport of doxorubicin

Because amifostine potentiated the activity of doxorubicin, we evaluated its potential effect on Pgp activity by flow cytometry. Results indicated that amifostine exerted no significant effect on the Pgp pump efflux activity (Table 1). CD34+ cells accumulated considerably less doxorubicin than normal lymphocytes, but this capacity was not influenced by amifostine at all doxorubicin concentrations tested.

### DISCUSSION

Preclinical studies in mice showed that amifostine pretreatment consistently decreases the toxic effect of radiation, nitrogen mustards, cisplatin, cyclophosphamide, carmustine, melphalan and 5-fluorouracil on haematopoietic progenitor cells (Wasserman et al, 1981). Recently, Shpall et al (1994) and Douay et al (1995) demonstrated that amifostine pretreatment protects human bone marrow CFU and long-term culture-initiating cells (LTC-IC) from mafosfamide or 4-hydroperoxycyclophosphamide (4-HC) treatment. Collectively, these data suggest that amifostine exerts a significant myeloprotective effect from toxicity produced by radiation, 5-fluorouracil and alkylating agents. These results were confirmed in three clinical studies in which amifostine significantly reduced the haematological toxicity of cyclophosphamide, carboplatin or cisplatin and cyclophosphamide (Glover et al, 1986; Betticher et al, 1995; Kemp et al, 1996). In this study, we tested the effectiveness of amifostine pretreatment in protecting unfractionated and purified human haematopoietic progenitors exposed to carboplatin, etoposide, doxorubicin and taxotere.

Unfractionated progenitor cultures in semisolid medium showed that a consistent and significant protective effect is produced by amifostine pretreatment on carboplatin, etoposide and high-dose taxotere (250 nM, which corresponds to the plasma peak levels achievable in vivo administering 70 mg $m^{-2}$ of drug; Bisset et al, 1993) exposed progenitors. Surprisingly, amifostine pretreatment significantly worsened the myelotoxic effect of doxorubicin. These results suggest that chemoprotection by amifostine is a drug-dependent process rather than an indefinite optimization of growth of those haematopoietic progenitors which escaped the toxic damage produced by the different agents. Liquid cultures of PB CD34+ cells confirmed that a significant chemoprotection can be produced by amifostine in etoposide- and carboplatin-treated progenitors. The detrimental effect exerted by amifostine on doxorubicin-exposed progenitors was documented also in this model of in vitro progenitor growth. This detrimental effect became evident from the 5th day of liquid culture onward with an evident decline of cell growth compared with doxorubicin alone, suggesting that the enhancement of doxorubicin toxicity by amifostine was the result of a progressive inability of cells to adequately proliferate in response to growth factor stimulation during culture rather than a greater cell killing immediately after drug exposure. Liquid cultures made possible the monitoring of apoptotic events, cycling status and viable cell replication rate (by progressive halving of a fluorescent dye) during etoposide-, carboplatin- and doxorubicin-exposed PB CD34+ cell growth in vitro after amifostine pretreatment, as well as in controls. The body of these data suggests that in etoposide- and carboplatin-treated progenitors amifostine pretreatment does not considerably affect the number of apoptotic events, the cell distribution into the different phases of the cell cycle and the number of cell divisions of viable cells from day 3 of culture onward. This fact suggests that amifostine produces a specific protection from etoposide and carboplatin toxic effects, increasing the fraction of cells which survive the cell damage generated by these drugs. Progenitor rescue after drug treatment could be mediated by the previously documented ability of amifostine to bind the active species of platinum agents, to reverse platin–DNA adduct formation and to repair DNA by hydrogen atom transfer or oxygen depletion (Purdie and Inhaber, 1983; Willson et al, 1983; Treskes and van der Vijgh, 1993). Conversely, in doxorubicin-exposed PB CD34+ progenitors, amifostine pretreatment produces an accumulation of cells in the $G_2/M$ phase of the cell cycle in the absence of a proportional increase in the viable cell division number, and in the presence of an increased number of dead cells in the cultures. This fact suggests that amifostine enhances the capacity of doxorubicin to produce cell death through a $G_2/M$ arrest, which is a well-known dose-dependent cytokinetic effect of anthracyclines (Bartkowiak et al, 1992). This study also shows that doxorubicin potentiation by amifostine is not mediated by an increased retention of doxorubicin, a possibility which must be excluded because haematopoietic progenitors are characterized by an active Pgp-mediated efflux system (Chaudhary and Roninson, 1991). On the whole, these data support the hypothesis that the potentiation of doxorubicin haematopoietic toxicity by amifostine in vitro could lie in the increased intracellular availability of free exogenous thiols which are known to catalyse the generation of hydrogen peroxide and hydroxyl radicals in the presence of a doxorubicin–iron complex (Muindi et al, 1985), or alternatively in the reported capacity of amifostine to reduce intracellular glutathione levels (Issels and Nagele, 1989; Meier and Issels, 1995). Using liquid cultures of PB CD34+ cells, we found that pretreatment with *N*-acetylcysteine produces an increase of the toxic effect of doxorubicin as well as amifostine does. The antioxidant nature of both compounds confirms that the detrimental effect on cell growth is probably due to a greater intracellular availability of exogenous antioxidant compounds. Therefore, our data suggest caution in the combined use of amifostine and doxorubicin until their pharmacological interaction can be fully clarified. Preliminary data from our laboratory indicate that amifostine does not worsen the myelotoxic effect of epirubicin, daunorubicin and mitoxantrone on purified PB CD34+ cells, and these preliminary findings are in harmony with the observations made by List et al (1996). Finally, our in vitro data on amifostine haematoprotection after treatments with etoposide, carboplatin and taxotere and the previously reported works on the chemoprotection of normal tissue after treatment with several chemotherapeutic agents encourage the clinical use of amifostine in preventing toxicity of anticancer therapy.

### REFERENCES

Bartkowiak D, Hemmer J and Rottinger E (1992) Dose dependence of the cytokinetic and cytotoxic effects of epirubicin in vitro. *Cancer Chemother Pharmacol* **30**: 189–192

© Cancer Research Campaign 1998

Betticher DC. Anderson H. Ranson M. Meely K. Oster W and Thatcher H (1995) Carboplatin combined with amifostine, a bone marrow protectant, in the treatment of non-small cell lung cancer: a randomized phase III study. *Br J Cancer* **72**: 1551–1555

Bissett D. Setanoians A. Cassidy J. Graham MA. Chadwick A. Wilson P. Auzannet V. Le Bail N. Kaye SB and Kerr DJ (1993) Phase I and pharmacokinetic study of taxotere (RP 56976) administered as a 24-hour infusion. *Cancer Res* **53**: 523–527

Calabro-Jones PM. Fahey RC. Smoluk GD and Ward JF (1985) Alkaline phosphatase promotes radioprotection and accumulation of WR-1065 in V79-171 cells incubated in medium containing WR-2721. *Int J Radiat Biol* **47**: 23–27

Calabro-Jones PM. Aguilera JA. Ward JF. Smoluk JD and Fahey RC (1988) Uptake of WR-2721 derivatives by cells in culture: identification of the transported form of the drug. *Cancer Res* **48**: 3634–3640

Chaudhary PM and Roninson IB (1991) Expression and activity of P-glycoprotein. a multidrug efflux pump. in human hematopoietic stem cells. *Cell* **66**: 85–94

Darzinkyewich Z. Juan G. Li X. Gorczyca W. Murakami T and Traganos F (1997) Cytometry in cell necrobiology: analysis of apoptosis and accidental cell death (necrosis). *Cytometry* **27**: 1–20

De Vincenzo R. Scambia G. Benedetti Panici P. Fattorossi A. Bonanno G. Ferlini C. Isola G. Pernisco S and Mancuso S (1996) Modulatory effect of tamoxifen and ICI 182.780 on adryamycin resistance in MCF-7 human breast-cancer cells. *Int J Cancer* **68**: 340–348

Douay L. Hu C. Giarratana M-C. Bouchet S. Conlon J. Capizzi RL and Gorin N-C (1995) Amifostine improves the antileukemic therapeutic index of mafosfamide: implications for bone marrow purging. *Blood* **86**: 2849–2855

Ferlini C. Biselli R. Nisini R and Fattorossi A (1995) Rhodamine 123: a useful probe to monitoring T cell activation. *Cytometry* **21**: 284–292

Glover DJ. Glick JH. Weiler C. Hurowitz S and Kligerman MM (1986) WR-2721 protect against the hematologic toxicity of cyclophosphamide: a controlled phase II trial. *J Clin Oncol* **4**: 584–588

Issels RD and Nagele A (1989) Promotion of cystine uptake, increase of glutathione biosynthesis, and modulation of glutathione status by *S*-2-(3-aminopropylamino)ethyl phosphorothioic acid (WR-2721) in Chinese hamster cells. *Cancer Res* **49**: 2082–2086

Kemp G. Rose P. Lurain J. Berman H. Mahetta A. Roullet B. Hamesley H. Belpomme D and Glick J (1996) Amifostine pretreatment for protection against cyclophosphamide and cisplatin-induced toxicities: results of a randomized controlled trials in patients with advanced ovarian cancers. *J Clin Oncol* **14**: 2101–2112

List AF. Heaton R. Glinsman-Gibson B and Capizzi RL (1996) Amifostine protects primitive hematopoietic progenitors against chemotherapy cytotoxicity. *Semin Oncol* **23**: 58–63

Lyons AB and Christopher RP (1994) Determination of lymphocyte division by flow cytometry. *J Immunol Methods* **171**: 131–137

Meier T and Issels RD (1995) Degradation of 2-(3-aminopropylamino)-ethanethiol (WR-1065) by Cu-dependent amine oxidases and influence on glutathione status of chinese hamster ovary cells. *Biochem Pharmacol* **50**: 489–496

Menichella G. Pierelli L. Scambia G. Salerno G. Benedetti Panici P. Foddai ML. Serafini R. Puglia G. Lai M. Ciarli M. Mancuso S and Bizzi B (1994) Low-dose cyclophosphamide in combination with cisplatin or epirubicin plus rhG-CSF allows adequate collection of PBSC for autotransplantation during adjuvant therapy for high-risk cancer. *Bone Marrow Transplant* **14**: 907–912

Muindi J. Sinha BK. Gianni L and Myers C (1985) Thiol-dependent DNA damage produced by anthracycline–iron complexes. The structure–activity relationships and molecular mechanisms. *Mol Pharmacol* **27**: 356–365

Ohnishi ST. Ohnishi T and Glick JH (1992) In vitro study on the antioxidant activities of amifostine (WR-2721) (abstract). *Proc Am Assoc Cancer Res* **33**: 419

Pierelli L. Menichella G. Paoloni A. Vittori M. Foddai ML. Serafini R. Rumi C. Mitschulat H. Rossi PL. Scambia G. Teofili L. Sica S. Leone G and Bizzi B (1993) Evaluation of a novel automated protocol for the collection of peripheral blood stem cells mobilized with chemotherapy or chemotherapy plus G-CSF using the Fresenius AS104 cell separator. *J Hematother* **2**: 145–153

Pierelli L. Scambia G. Menichella G. Fattorossi A. Ciarli M. Bonanno G. Battaglia A. d'Onofrio G. Benedetti Panici P. Iacone A. Mancuso S and Leone G (1997) Purified unfractionated G-CSF/chemotherapy mobilized CD34+ peripheral blood progenitors and not bone marrow CD34+ progenitors undergo selective erythroid differentiation in liquid culture in the presence of erythropoietin and stem cell factor. *Br J Haematol* **96**: 55–63

Purdie JW and Inhaber ER (1983) Interaction of cultured mammalian cells with WR-2721 and its thiol WR-1065: implications for mechanism of radioprotection. *Int J Radiat Biol* **43**: 517–527

Shpall EJ. Stemmer SM. Hami L. Franklin WA. Shaw L. Bonner HS. Bearman SI. Peters WP. Bast Jr RC. McCulloch W. Capizzi R. Mitchell E. Schein PS and Jones RB (1994) Amifostine (WR-2721) shortens the engraftment period of 4-hydroperoxycyclophosphamide-purged bone marrow in breast cancer patients receiving high-dose chemotherapy with autologous bone marrow support. *Blood* **83**: 3132–3137

Treskes M and van der Vijgh WJ (1993) WR2721 as a modulator of cisplatin- and carboplatin-induced side effects in comparison with other chemoprotective agaents: a molecular approach. *Cancer Chemother Pharmacol* **33**: 93–106

Wasserman TH. Phillips TL. Ross G and Kane LJ (1981) Differential protection against cytotoxic chemotherapeutic effects on bone marrow CFUs by WR-2721. *Cancer Clin Trials* **4**: 3–6

Willson RL (1983) Free radical repair mechanisms and interaction of glutathione and vitamins C and E. In *Radioprotectors and Anticarcinogenesis*. Nygaard OF and Simic MG (eds). pp. 1–22. Academic Press: New York

Yuhas JM (1969) Differential protection of normal and malignant tissues against the cytotoxic effects of mechloretamine. *Cancer Treat Rep* **63**: 971–976

Yuhas JM and Storer JB (1969) Differential chemoprotection of normal and malignant tissues. *J Natl Cancer Inst* **42**: 331–335

Yuhas JM. Spellman JM and Culo F (1980*a*) The role of WR-2721 in radiotherapy and/or chemotherapy. In *Radiation Sensitizer*. Brady L (ed.). pp. 303–308. Masson: New York

Yuhas JM. Spellman JM and Jordan SW (1980*b*) Treatment of tumours with the combination of Amifostine and *Cis*-Dichlorodiammineplatinum (II) or cyclophosphamide. *Br J Cancer* **42**: 574–585

© Cancer Research Campaign 1998